# Supplementary material for: Methane-cycling microbiomes in soils of the pan-Arctic and their response to permafrost degradation
Source: Commun Earth Environ. 2025 Sep 16;6(1):748. doi: 10.1038/s43247-025-02765-5 (PMC12440815; doi:10.1038/s43247-025-02765-5)
Supplement: Supplementary file 2 — Supporting Information [file 43247_2025_2765_MOESM2_ESM.pdf]

Supporting Information

Methane-cycling microbiomes in soils of the pan-Arctic and their response to permafrost degradation

Haitao Wang[1*], Erik Lindemann[1], Patrick Liebmann[2], Milan Varsadiya[3], Mette Marianne Svenning[4], Muhammad Waqas[3], Sebastian Petters[1,5], Andreas Richter[6], Georg Guggenberger[2], Jiri Barta[3], Tim Urich[1*]

1. Institute of Microbiology, University of Greifswald, Greifswald, Germany

2. Institute of Earth System Sciences, Section Soil Science, Leibniz Universität Hannover, Hannover, Germany

3. Department of Ecosystem Biology, University of South Bohemia, České Budějovice, Czech Republic

4. Department of Arctic and Marine Biology, The Arctic University of Norway, Tromsø, Norway

5. The Norwegian College of Fishery Science, The Arctic University of Norway, Tromsø, Norway

6. Centre for Microbiology and Environmental Systems Science, University of Vienna, Vienna, Austria

Correspondence to haitao.wang@uni-greifswald.de or tim.urich@uni-greifswald.de

Running head: Pan-Arctic methane-cycling microbiome

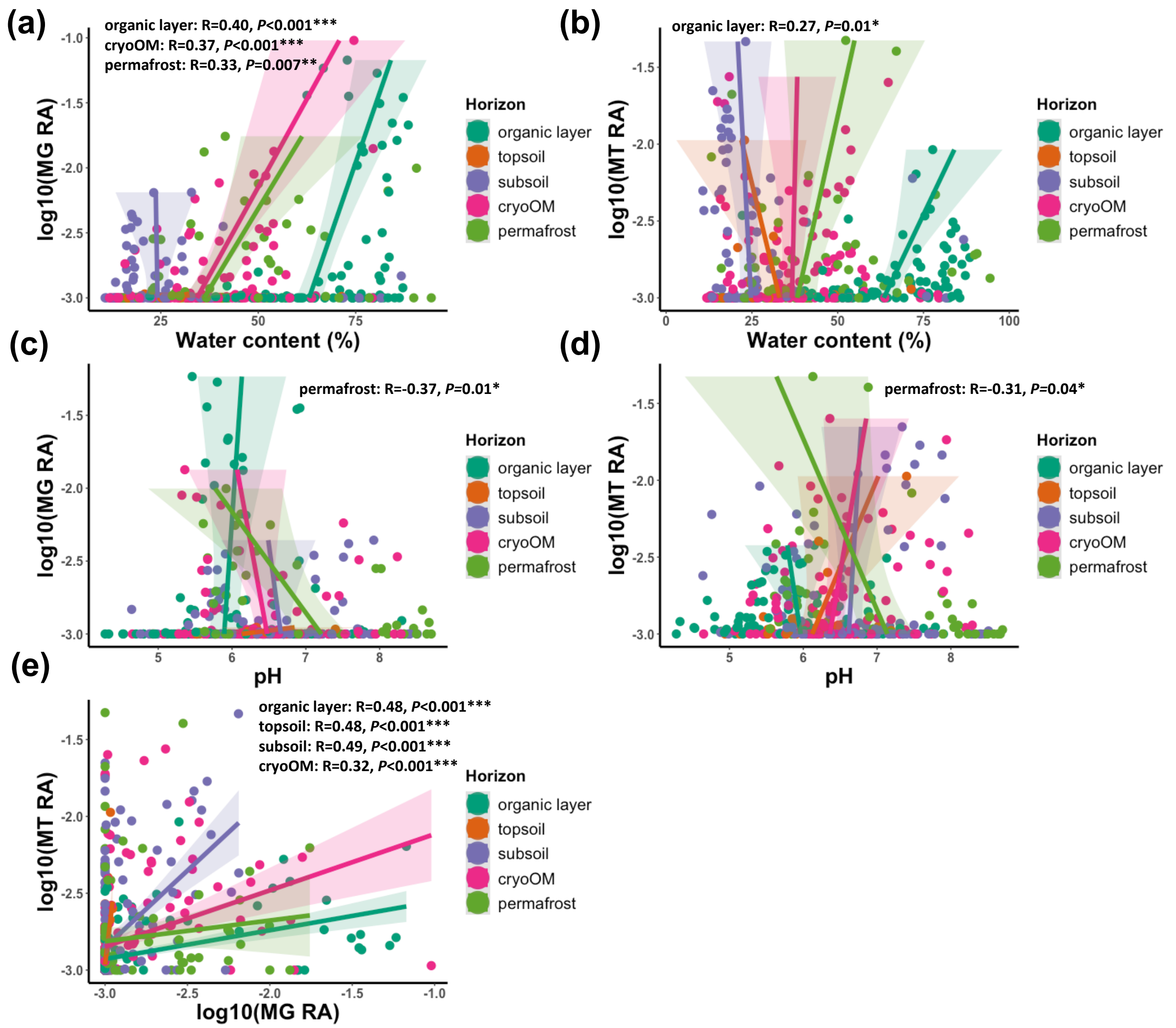

Fig. S1 Correlations between water content and (a) methanogen and (b) methanotroph abundances, correlations between pH and (c) methanogen and (d) methanotroph abundances, and (e) correlation between methanogen and methanotroph abundances. MG, methanogen; MT, methanotroph; RA, relative abundance. The R and *P* values are based on Pearson's correlation. A value of 0.001 was added to MG and MT relative abundances before $\log_{10}$ transformation to avoid zeros. The significant correlations were also confirmed as significant with Spearman's correlation. Only statistics (R and *P* values) with a significant correlation are shown.

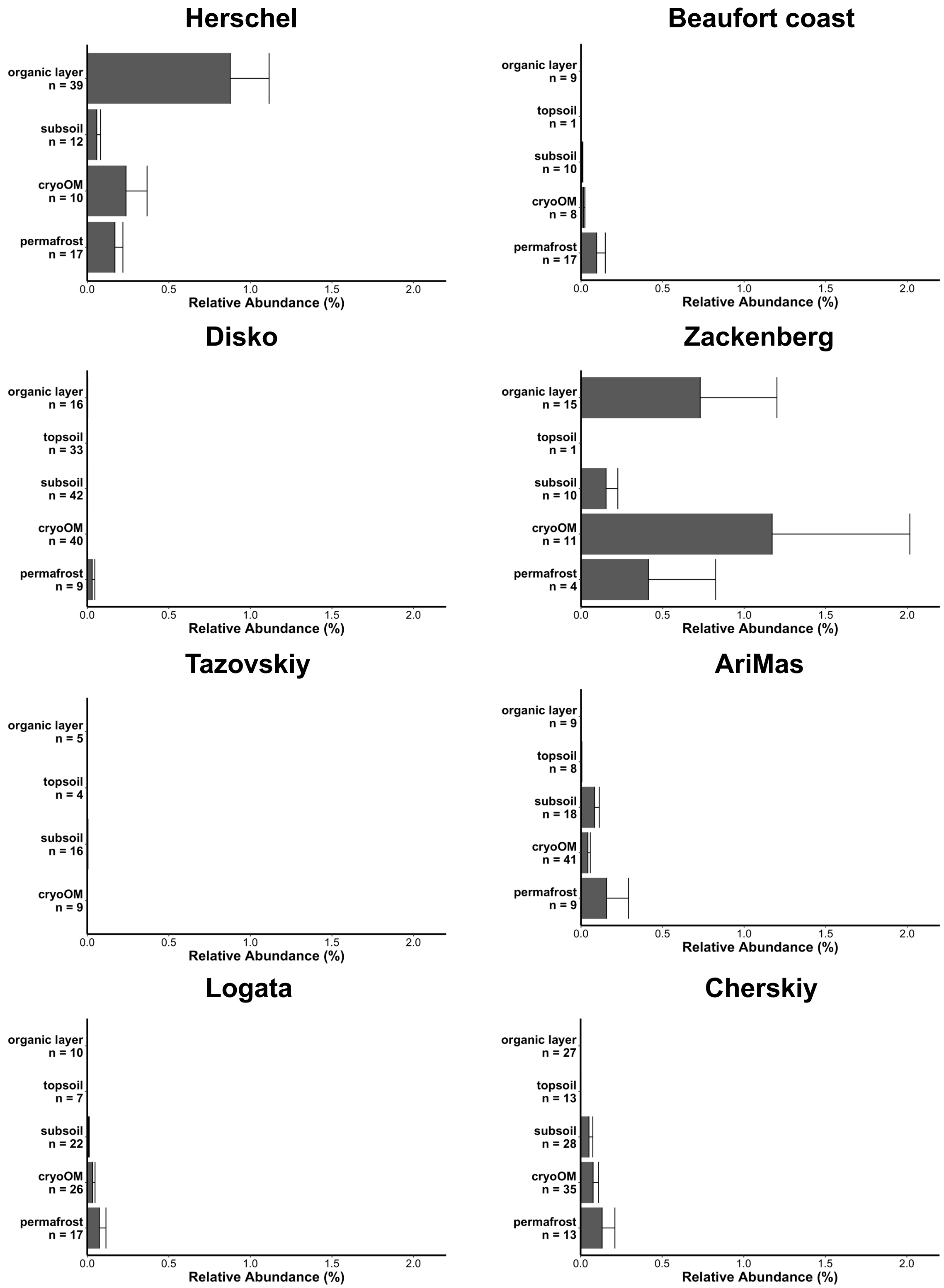

Fig. S2 Relative abundance of methanogens in different horizons at each site. Abundances are shown as mean + standard error.

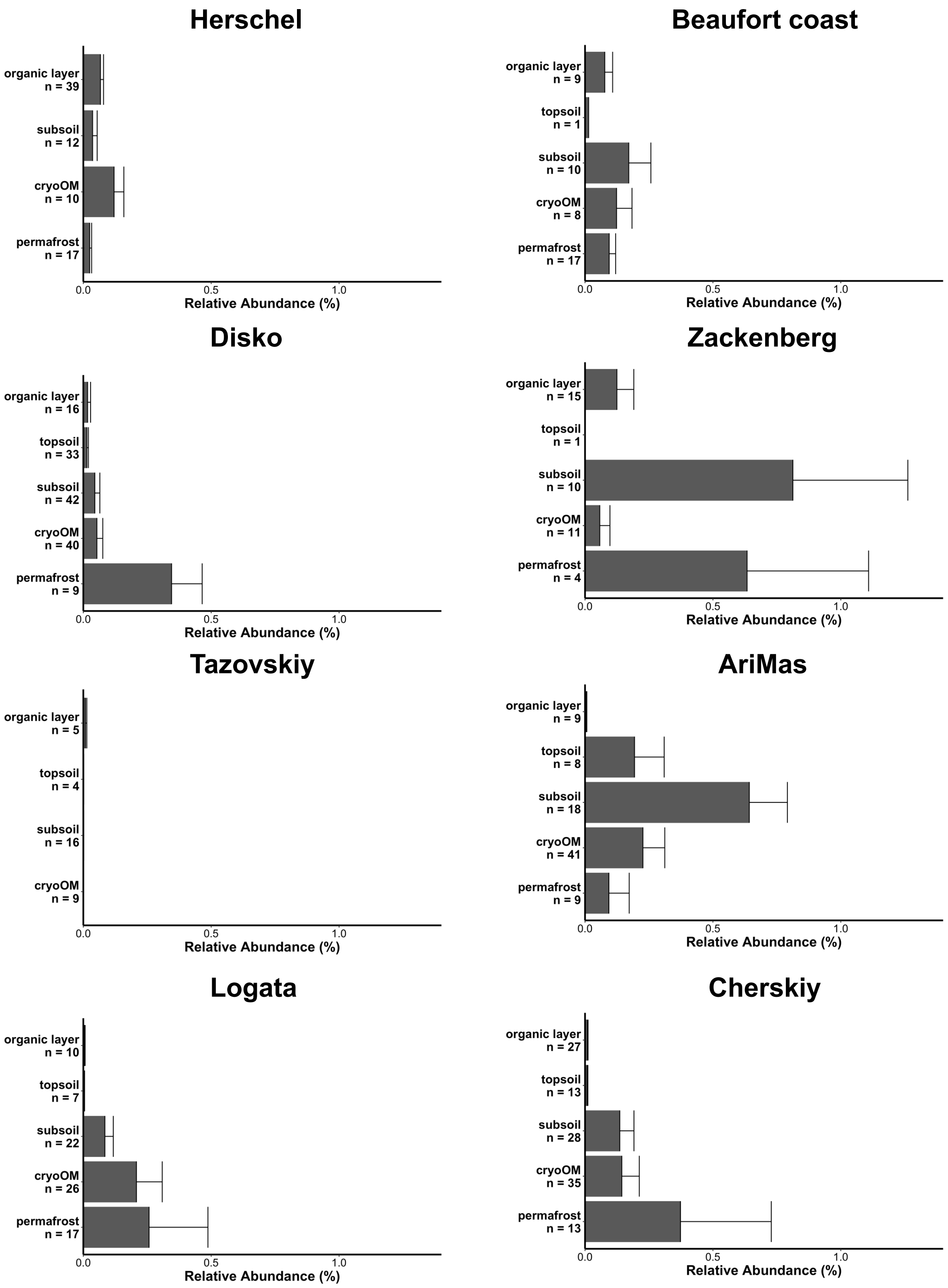

Fig. S3 Relative abundance of methanotrophs in different horizons at each site. Abundances are shown as mean + standard error.

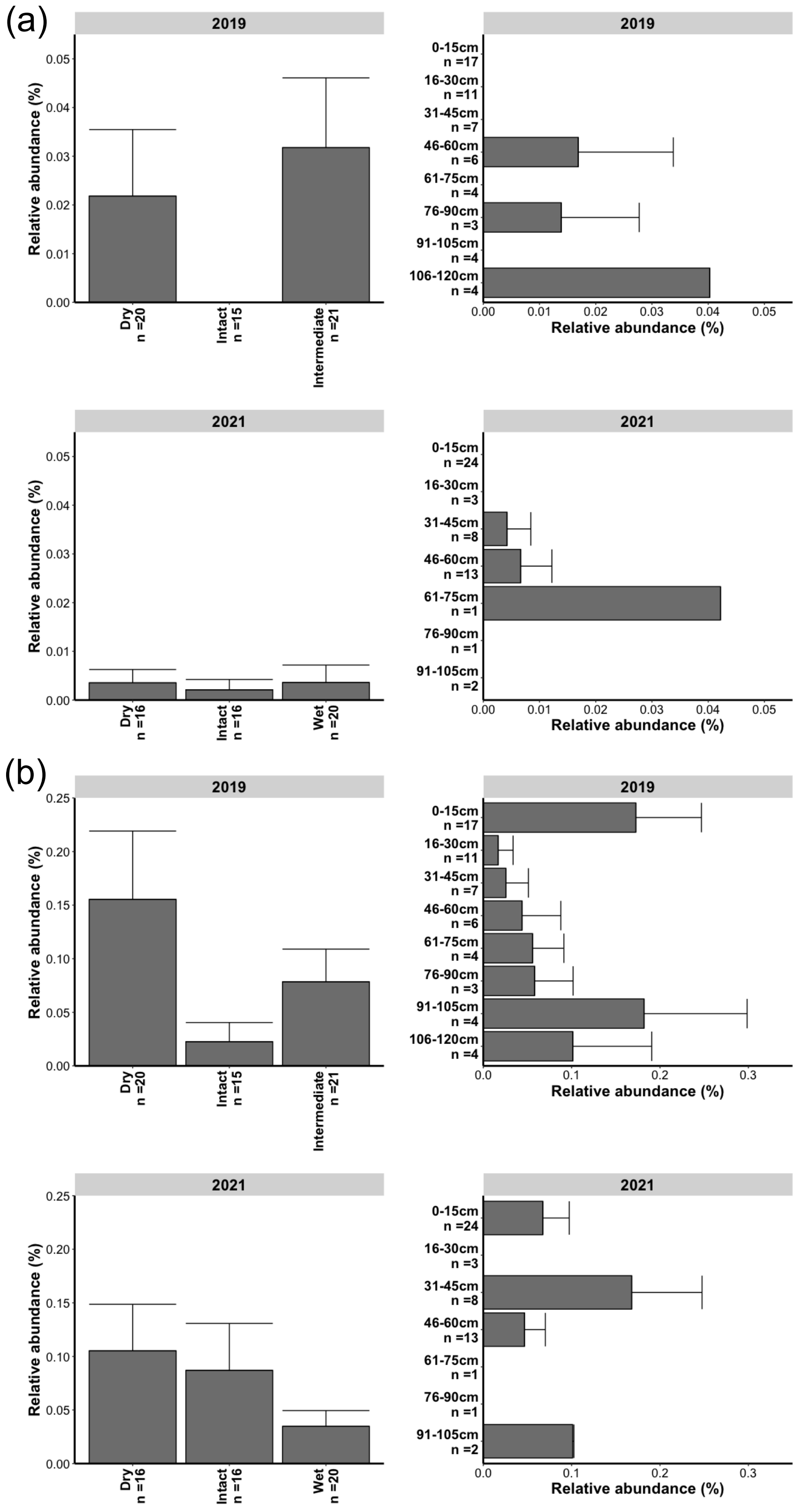

Fig. S4 Relative abundances of (a) methanogens and (b) methanotrophs in between different scenarios and depths in 2019 and 2021. Abundances are shown as mean + standard error. No

significant difference was detected ($P > 0.05$) based on Kruskal-Wallis *post hoc* Dunn's tests with P values adjusted by the false discovery rate method.

Table S1 Taxonomy list used for identifying methanogens and methanotrophs based on 16S rRNA gene taxonomy

	Phylum	Class	Order	Family	Genus	No. of ZOTUs
**Methanogens**	Euryarchaeota	Methanobacteria	**Methanobacteriales**	-	-	8
	Euryarchaeota	Methanopyri	**Methanopyrales**	-	-	-
	Euryarchaeota	Methanococci	**Methanococcales**	-	-	-
	Methanobacteriota	Thermococci	**Methanofastidiosales**	-	-	-
	Halobacteriota	Methanocellia	**Methanocellales**	-	-	-
	Halobacteriota	Methanomicrobia	**Methanomicrobiales**	-	-	6
	Halobacteriota	Methanosarcinia	**Methanosarciniales** (excluding ANME)	-	-	6
	Halobacteriota	**Methanonatronarchaeia**	-	-	-	-
	Thermoplasmatota	Thermoplasmata	**Methanomassiliicoccales**	-	-	2
**Methanotrophs**	Proteobacteria	Alphaproteobacteria	Rhizobiales	Beijerinckiaceae	**Methylocystis**	-
	Proteobacteria	Alphaproteobacteria	Rhizobiales	Beijerinckiaceae	**Methylosinus**	-
	Proteobacteria	Alphaproteobacteria	Rhizobiales	Beijerinckiaceae	**Methylocapsa**	-
	Proteobacteria	Alphaproteobacteria	Rhizobiales	Beijerinckiaceae	**Methyloferula**	1
	Proteobacteria	Alphaproteobacteria	Rhizobiales	Beijerinckiaceae	**Methylocella**	1
	Proteobacteria	Gammaproteobacteria	**Methylococcales**	-	-	21
	Verrucomicrobiota	Verrucomicrobiia	Methylacidiphilales	Methylacidiphilaceae	**Methylacidiphilum** **Methylacidimicrobium**	-
	Methylomirabilota	Methylomirabilia	Methylomirabilales	Methylomirabilaceae	**Ca. Methylomirabilis**	1
	Halobacteriota	Methanosarcinia	Methanosarciniales	**ANME-2a, 2b and 2c**	-	-
	Halobacteriota	Methanosarcinia	Methanosarciniales	**Methanoperedenaceae (ANME-2d)**	-	2
	Halobacteriota	**ANME-1**	-	-	-	-

This list is based on SILVA v138.2

Table S2 Matching ASVs to ZOTUs with a 100% identity using BLASTN

ASV	ASV taxonomy	ZOTU (100% identity)	ZOTU taxonomy
ASV9819	Methylomonadaceae:Methylobacter	ZOTU3405	Methylomonadaceae:Methylobacter
ASV9660	Methylomonadaceae:Methylobacter	ZOTU1199	Methylomonadaceae:Methylobacter
ASV8822	Methylomonadaceae:Methylobacter	ZOTU366/1239	Methylomonadaceae:Methylobacter
ASV7750	Methylomonadaceae:Methylobacter	ZOTU1727/1519	Methylomonadaceae:Methylobacter
ASV7357	Methylomonadaceae:Methylobacter	ZOTU1199	Methylomonadaceae:Methylobacter
ASV6940	Methylomonadaceae:Methylobacter	ZOTU366/1239	Methylomonadaceae:Methylobacter
ASV6209	Methylomonadaceae:Methylobacter	NA	
ASV6205	Methylomonadaceae:Methylobacter	ZOTU1253	Methylomonadaceae:Methylotuvimicrobium
ASV6061	Methylomonadaceae:Methylobacter	ZOTU1199	Methylomonadaceae:Methylobacter
ASV5398	Methylomonadaceae:Methylobacter	ZOTU366/1239	Methylomonadaceae:Methylobacter
ASV4596	Methylomonadaceae:Methylobacter	ZOTU1199	Methylomonadaceae:Methylobacter
ASV11464	Methylomonadaceae:Methylobacter	ZOTU1253	Methylomonadaceae:Methylotuvimicrobium
ASV11262	Methylomonadaceae:Methylobacter	ZOTU366/1239	Methylomonadaceae:Methylobacter
ASV7590	Methylomonadaceae:Methylobacter	ZOTU4804	Methylomonadaceae:Methylobacter
ASV3295	Methylomonadaceae:Methylobacter	ZOTU4804	Methylomonadaceae:Methylobacter
ASV2044	Methylomonadaceae:Methylobacter	ZOTU3405	Methylomonadaceae:Methylobacter
ASV11037	Methylomirabilaceae:Ca. Methylomirabilis	ZOTU3370	Methylomirabilaceae:Ca. Methylomirabilis
ASV10087	Methylomirabilaceae:Ca. Methylomirabilis	ZOTU3370	Methylomirabilaceae:Ca. Methylomirabilis
ASV9046	Beijerinckiaceae:Methylocapsa	NA	
ASV8524	Beijerinckiaceae:Methylocapsa	NA	
ASV7447	Beijerinckiaceae:Methylocapsa	NA	
ASV7181	Beijerinckiaceae:Methylocapsa	NA	
ASV5264	Beijerinckiaceae:Methylocapsa	NA	
ASV469	Beijerinckiaceae:Methylocapsa	NA	
ASV3351	Beijerinckiaceae:Methylocapsa	NA	
ASV2716	Beijerinckiaceae:Methylocapsa	NA	
