# Supplementary material for: Methane-cycling microbiomes in soils of the pan-Arctic and their response to permafrost degradation
Source: Commun Earth Environ. 2025 Sep 16;6(1):748. doi: 10.1038/s43247-025-02765-5 (PMC12440815; doi:10.1038/s43247-025-02765-5)
Supplement: Supplementary file 3 — Description of Additional Supplementary Files [file 43247_2025_2765_MOESM3_ESM.pdf]

Description of Additional Supplementary Files

File name: Supplementary Data 1
Description: Tables showing the taxonomy of each phylotype and their representative sequences. The four tables present information of methanogen and methanotroph phylotypes from the pan-Arctic dataset (ZOTUs) and Alaksa dataset (ASVs), respectively. Each table also shows the identity of the best match based on NCBI BLASTN against the 16S rRNA gene database. Some taxonomy identifications were fixed based on the BLASTN results which are highlighted in blue in each table.
